# Parental knowledge, awareness, and attitudes towards children’s oral habits: a descriptive cross-sectional study

**DOI:** 10.2340/aos.v84.42643

**Published:** 2025-01-30

**Authors:** Sarah AlMugairin, Alanoud Alwably, Nada Alayed, Alanoud Algazlan, Hadeel Alrowaily, Elzahraa Eldwakhly, Abdullazez Almudhi

**Affiliations:** a Department of Preventive Dental Sciences, College of Dentistry, Princess Nourah bint Abdulrahman University, P.O. Box 84428, Riyadh 11671, Saudi Arabia; b College of Dentistry, Princess Nourah Bint Abdulrahman University, Riyadh, Saudi Arabia; c Department of Oral medicine and pathology, College of Dentistry, King Saud University, Riyadh, Saudi Arabia; d Department of Oral and maxillofacial surgery, Security forces hospital, Riyadh, Saudi Arabia; e Department of Clinical Dental Sciences, College of Dentistry, Princess Nourah bint Abdulrahman University, P.O. Box 84428, Riyadh 11671, Saudi Arabia; f Department of Pediatric Dentistry and Orthodontics, College of Dentistry, King Saud University, Riyadh, Saudi Arabia

**Keywords:** Oral habits, prevention, parental awareness, pediatric dentistry, pacifier, behavior guidance

## Abstract

**Background:**

Oral health is fundamental to children’s health and well-being. Parental knowledge, awareness, and practices towards oral habits significantly influence children’s oral health. Early diagnosis and intervention to break abnormal oral habits are vital to prevent long-term detrimental effects on oral and facial development. Objective: This study aimed to assess parents’ knowledge, awareness, and attitudes towards their children’s oral habits in Riyadh, Saudi Arabia. Methods: A cross-sectional study design was employed, where 2,000 participants were enrolled, of whom 563 Saudi mothers residing in Riyadh met the inclusion criteria. A validated, self-administered questionnaire was used to collect data on demographics, child information, parental awareness, and parental attitudes.

**Results:**

Regarding awareness, moderate overall awareness was reported among mothers, with the majority (over 70%) correctly identifying the negative effects of prolonged pacifier/bottle use and other habits on children’s oral health. As for the attitudes, most mothers recognized the importance of stopping sucking habits (digit and/or pacifier) by 18 months and agreed that persistent oral habits cause malocclusion and growth problems. However, a significant gap existed between knowledge and practice, with most mothers (92.9%) never consulting a dentist regarding their child’s ongoing oral habits. Mothers with higher education levels reported encouraging their children to stop habits and documented improvements observed after habit cessation.

**Conclusions:**

Although most mothers demonstrated moderate awareness of the detrimental effects of prolonged oral habits, a clear gap remains between knowledge and practice. This study emphasizes the need for educational programs to improve parental knowledge, attitudes, and practices regarding children’s oral habits. Additionally, addressing cultural beliefs and cost barriers to dental treatment could increase the utilization of dental services, improving children’s oral health.

## Introduction

Children’s oral health plays a crucial role in laying down the foundation for healthy permanent teeth. Parents’ knowledge and attitudes significantly impact the maintenance of oral health in young children [1, 2]. Raising public awareness of children’s oral health is essential to achieving effective oral health preservation [3, 4].

Infant oral health care lays the foundation for lifelong preventive education and dental care, promoting optimal oral health into childhood and adulthood [1, 5]. Contrary to the misconception that primary teeth are temporary, they are essential for overall health, mastication, speech, aesthetics, and self-esteem, greatly influencing the quality of life [6, 7].

Early dental visits establish a positive child–dentist relationship and educate parents on preventive care, forming the foundation for preventive dental care [8]. The American Academy of Pediatric Dentistry (AAPD) and the American Dental Association (ADA) recommend the first dental visit within 6 months of the first tooth eruption or by age one [9, 10]. Tooth brushing with fluoridated toothpaste twice daily and limiting snacks are advised to promote good oral habits [11, 12]. This period is also ideal for discussing healthy habits and evaluating behaviours’ risks and benefits [13, 14].

A habit is an action acquired through frequent repetition, initially conscious and later unconscious [15]. Oral habits are classified as normal or parafunctional. Normal physiological and functional habits, like nasal breathing, chewing, and swallowing, support normal occlusion and facial growth [16, 17]. Parafunctional habits are abnormal motor activities of the masticatory system, reflecting disharmony with the environment. [18]. Persistent abnormal habits in children aged 4–6 years can cause dentofacial discrepancies, and the AAPD recommends discontinuing non-nutritive sucking, such as pacifier use, by the age of 36 months [3, 19].

Oral parafunctional habits include biting activities (e.g. nail-biting), bruxism (teeth clenching), and soft tissue activities (e.g. thumb and pacifier sucking, tongue thrusting, and lip biting). These habits are classified as occlusal (involving teeth contact) or non-occlusal (e.g. biting labial and buccal mucosa) [18, 20]. For years, oral habits in infants and young children have been debated among parents, pediatricians, psychologists, speech pathologists, and pediatric dentists. Early diagnosis of abnormal habits enables intervention from both dentists and parents to prevent negative outcomes [21–23].

Oral habits may be harmful depending on factors such as duration, frequency, and intensity, as well as genetic predispositions, which influence facial, occlusal, and muscular changes [24, 25]. Persistent parafunctional habits can stem from emotional issues such as insecurities loneliness or neglect experienced by the child [17, 24]. In contrast, non-nutritive sucking (e.g. finger or pacifier sucking) in children up to 36 months is considered normal and satisfies the need for contact and security [17, 26]. However, the persistence of parafunctional habits risks dentofacial development disruptions and malocclusions [27, 28].

These habits cause malocclusions such as proclined and retroclined incisors, increased overjet, decreased overbite, posterior crossbite, or open bite [29], and are linked to deviated osseous growth, tooth malposition, breathing and speech issues, imbalanced facial musculature, and/or psychological problems [27, 29].

A 2018 study by Aloumi et al. reported nail-biting as the most prevalent habit (27.2%) among preschool children in Riyadh, followed by mouth breathing (13.8%), thumb sucking (7.4%), and teeth clenching (6.0%) [18]. A recent study on on children aged 7–13 found similar results, with nail-biting being the most common habit (24.2%), while sleep bruxism had the lowest prevalence (6.6%), indicating relationships between harmful oral habits and malocclusions [30].

A study by Fernandes and Lima 2019, found that children not adequately breastfed in the first 6 months are more likely to develop parafunctional habits [31]. Prolonged bottle-feeding can affect sucking, swallowing, and chewing, leading to malocclusions [29]. Aloumi et al. 2018, reported a significant correlation between malocclusion and thumb-sucking or pacifier-sucking habits [18].

Factors such as high dental costs, low socioeconomic status, lower parental education, and lack of awareness of paediatric dentists affect the timing of a child’s first dental visit [32]. Parents with higher education are more proactive and committed to their child’s oral health [33]. Lower education and income levels negatively impact parents’ knowledge and awareness of children’s oral health [34–36].

Good oral health is crucial for overall health and well-being. Children’s oral health depends significantly on parents’ awareness, knowledge, education, and socioeconomic status [37]. This study examines parents’ knowledge, awareness, and attitudes regarding their children’s oral habits in Riyadh, Saudi Arabia.

## Materials and methods

### Study design

This study employed a cross-sectional, observational design to assess the knowledge, awareness, and attitudes of Saudi mothers residing in Riyadh, Saudi Arabia, regarding their children’s oral habits.

### Participants selection criteria

#### Inclusion criteria

Saudi mothers aged 20–50 years.

Mothers with at least one child between the ages of two and 12 (inclusive).

This study included only mothers with children over the developmental age of two, as this age marks a transition where normal, comforting developmental habits such as thumb-sucking or pacifier use if prolonged beyond infancy, can become potentially harmful and lead to malocclusions affecting normal oral development, as reported by several studies [16, 31]. By age two, children are typically transitioning away from behaviors such as breastfeeding or bottle-feeding, and it becomes more relevant to monitor non-nutritive sucking habits such as pacifier or thumb sucking, which can impact dental development if prolonged. Thus, including only children older than two ensures that the study focuses on clinically relevant habits for their potential impact on oral health and development. This is in alignment with recommendations of the American Academy of Pediatric Dentistry of discontinuing non-nutritive sucking habits like pacifier use by age three to prevent dental malocclusion and other issues [9].

#### Exclusion criteria

Non-Saudi mothers

 Mothers with children younger than 2 years or older than 12

 Mothers of children with special healthcare needs

#### Dropout criteria

Participants who completed only part of the questionnaire were considered dropouts.

### Sample size

The study enrolled 2,000 participants who signed the consent form. The sample size calculation was based on a previous study [38]. Considering a 95% confidence interval, a 5% margin of error, and considering potential non-responses, an actual sample size of 563 mothers was recruited for the study.

### Data collection

Data were collected using a self-administered questionnaire developed and validated to assess participants’ knowledge, awareness, and attitudes regarding their children’s oral habits. Two dentists who were fluent in English and Arabic designed the questionnaire using an online survey platform in English; the survey was then translated into Arabic for the participants’ convenience. The Arabic version was then back-translated into English by another two people fluent in Arabic and English. Finally, the back-translated version was compared with the English version to verify proper translation.

The inter-translator agreement process involved translating the questionnaire from English to Arabic and then back-translating it to English. The original and back-translated English versions were compared to ensure that the Arabic translation accurately preserved the original same content’s meaning. The two independent forward translations showed complete agreement, with no discrepancies identified between the versions produced by the translators. This process helped ensure the reliability and accuracy of the questionnaire in both languages.

The questionnaire was divided into four sections:

1. Demographic Data: Questions about mothers (age, nationality, education, socioeconomic status, occupation, and number of children).
2. Child Information: Questions about the child’s age, gender, medical conditions, bottle or breastfeeding, weaning age, use of a pacifier, length of use of a pacifier, and adoption of any oral habits.
3. Parental Awareness: Questions assessing mothers’ knowledge about the types and consequences of common oral habits in children.
4. Parental Attitudes: Questions evaluating mothers’ attitudes towards preventing and managing oral habits in their children.

### Ethical approval

Ethical approval to conduct the study was obtained from the Institutional Review Board of the College of Dentistry at Princess Nourah Bint Abdulrahman University (protocol code H-01-R-059, 24-11-2020). Informed consent was obtained from all participants before completing the questionnaire and commencing the study. It was clarified to the participants at the beginning of the questionnaire that participation in this study was voluntary, and that the anonymity and confidentiality of the responses were assured.

### Statistical analysis

Descriptive statistics (frequency and percentages) were used to summarise and describe the participants’ demographic characteristics, and knowledge, awareness, and attitude scores. Pearson’s Chi-squared test was used to compare the responses of knowledge, awareness, and attitude items on oral habits, and to compare the distribution across the categorical study variables (e.g. educational status and number of children). A *p*-value of ≤ 0.05 was used to report the statistical significance of results. Data were analysed using the Statistical Package for the Social Sciences, SPSS 26.0 statistical software (IBM, Inc., Chicago, USA).

### Expected outcome

Given the limited data available on parental knowledge regarding children’s oral habits in Saudi Arabia, this study was anticipated to highlight potential gaps in parental awareness regarding proper identification and management of parafunctional oral habits in children. This study aimed to raise parental awareness concerning interventional measures to improve oral health practices among children.

## Results

### Demographics and child characteristics, Table 1

A total of 563 mothers participated in this study. Table 1 summarises participating mothers’ demographics. Most of the mothers (42.5%) were aged between 31 and 40. Most participating mothers (71.2%) held a Bachelor’s degree. A monthly income of 10 to 15 thousand Saudi Riyals was reported by 34.8% of the mothers, while nearly half (45.8%) were unemployed. An income of 10,000 to 15,000 Saudi Riyals is equivalent to an income of approximately 2,700 USD to 4,000 USD. The number of children per mother was evenly distributed across the three identified categories (1–2 years old), (3–4 years old) and (5+ years old). Over half of the children were females.

**Table 1:** Socio-demographic characteristics of study subjects.

Characteristics	No. (%)
**Mother’s Age (in years)**	
20–30	53 (9.4)
31–40	239 (42.5)
41–50	191 (33.9)
51–60	80 (14.2)
**Mother’s Education**	
Lower than High school	34 (6.0)
High School	79 (14.0)
Bachelors/Diploma	401 (71.2)
PG (Master’s or PhD)	49 (8.7)
**Family socio-economic status**	
No income	14 (2.5)
< 5,000	27 (4.8)
5,000–10,000	101 (17.9)
10,000–15,000	196 (34.8)
15,000–20,000	85 (15.1)
20,000–25,000	40 (7.1)
> 25,000	100 (17.8)
**Mother’s occupation**	
Unemployed status	258 (45.8)
Governmental employee	220 (39.1)
Private employee	46 (8.2)
Both Governmental and Private employee	10 (1.8)
Others	29 (5.2)
**Number of children**	
1–2	176 (31.3)
3–4	175 (31.1)
5 or more	212 (37.7)
**Child age**	
2–4	203 (36.1)
5–7	90 (16.0)
> 8	270 (48.0)
**Child gender**	
Male	269 (47.8)
Female	294 (52.2)

The results of this study elaborated on the associations between maternal demographics (such as education level, socioeconomic status, number of children) and dental knowledge and practices regarding children’s oral health. Higher maternal education is linked to better awareness and proactive behaviours, such as encouraging children to stop harmful oral habits and seek professional consultation. The results also proved that socioeconomic status did impacts dental care access and parental awareness, with higher-income families generally showing more positive oral health behaviours. These associations emphasise the need for targeted educational programmes to address gaps in parental knowledge, particularly in lower socioeconomic and educational groups.

### Children’s feeding practices and oral habits, Table 2

Approximately, 60% of the children received both breast and bottle-feeding. Weaning occurred around 2 years of age for 45.6% of those children. Pacifier use was prevalent in 45.3% of children and over 60% stopped such use between the ages of 3–6 years. A total of 229 children (40%) were reported to exhibit oral habits. Nail-biting (25.5%) was the most prevalent oral habit among these children with reported oral habits, followed by teeth clenching (17.1%), finger sucking (10.5%); 9.8% adopted other habits.

**Table 2:** Distribution of variables related to child’s feeding and oral habits.

Variables of feeding and oral habits	No. (%)
**Type of feeding**	
Breast	80 (14.2)
Bottle	143 (25.4)
Both	340 (60.4)
**Age of child weaned (in years)**	
< 1	132 (23.4)
1	64 (11.4)
2	257 (45.6)
3	67 (11.9)
> 3	18 (3.2)
Did not wean my child yet	25 (4.4)
**Child use a pacifier**	
Yes	255 (45.3)
No	308 (54.7)
**If yes, age of child stop using the pacifier (in years)**	
< 1	10 (3.9)
1–2	87 (34.1)
3–4	109 (42.7)
5–6	49 (19.2)
**Type of Child’s habits (multiple responses) (*n* = 229)**	
Digit (finger)sucking	29 (10.5)
Tongue thrusting	2 (0.7)
Mouth breathing	27 (9.8)
Lip/cheek biting	10 (3.6)
Teeth clenching	47 (17.1)
Nail Biting	70 (25.5)
I don’t know	63 (22.9)
Other habits	27 (9.8)

### Parental knowledge regarding children’s oral habits, Table 3

Table 3 presents the results assessing mothers’ knowledge regarding their children’s oral habits. The comparison of parental knowledge responses about their children’s oral habits revealed statistically significant differences (*p* < 0.0001) for all questions assessing mothers’ knowledge about the types and consequences of common oral habits in children.

**Table 3:** Distribution and comparison of parental knowledge responses towards their children’s oral habits.

Items of knowledge	No. (%)	Χ^2^-value	*p*
Do you think prolonged use of pacifiers or bottle feeding have an effect on your child’s teeth?		288.27	< 0.0001
Yes	375 (66.6)		
No	67 (11.9)		
I don’t know	121 (21.5)		
Do you think that some oral habits such as (digit sucking, lip and cheek biting) can affect your child teeth?		436.84	< 0.0001
Yes	420 (74.6)		
No	49 (8.7)		
I don’t know	94 (16.7)		
Do you think oral habits can be prevented?		487.74	< 0.0001
Yes	432 (76.7)		
No	34 (6.0)		
I don’t know	97 (17.2)		
At what age do you think it is recommended to help a child stop a sucking habit (such as pacifier or digit sucking)		462.88	< 0.0001
18 months	421 (74.8)		
3 years	122 (21.7)		
6 years	20 (3.6)		
Which of the following problems can be caused by persistent oral habits? (Multiple responses) (*n* = 522)		-	-
Dental cavities	123 (12.2)		
Malocclusion (badly aligned teeth)	311 (30.7)		
Teeth discoloration	30 (3.0)		
Speech problems	137 (13.5)		
Early loss of teeth	33 (3.3)		
Aesthetic problems	95 (9.4)		
Growth problems of the jaws	191 (18.9)		
No effect	92 (9.1)		
Which aspect regarding oral habits do you think is more important and can have an effect on teeth and facial changes?		46.31	< 0.0001
Frequency of the habit	129 (22.9)		
Duration of the habit per day	175 (31.1)		
Intensity of the force and pressure present during the habit	259 (46.0)		
Do you think the habit of breaking treatment will be different according to the child’s age?		729.02	< 0.0001
Yes	488 (86.7)		
No	10 (1.8)		
I don’t know	65 (11.5)		

For the question ‘Do you think prolonged use of pacifiers or bottle feeding affects your child’s teeth?’, the majority (66.6%) correctly identified prolonged pacifier/bottle use as detrimental, whereas 21.5% of participating mothers responded, ‘I do not know’.

Similarly, 74.6% and 76.7% of mothers responded ‘Yes’ to the questions ‘Do you think that some oral habits such as digit sucking, lip, and cheek biting can affect your child’s teeth?’ and ‘Do you think oral habits can be prevented?’, respectively, with high statistical significance (*p* < 0.0001 for both). Most mothers (over 70%) believed children should stop sucking habits by 18 months.

Notably, 46% of mothers recognised the importance of force and pressure on teeth during oral habits as the key factor influencing teeth and facial development. Additionally, 86.7% agreed that treatments for breaking habits varied according to the child’s age.

Regarding potential problems from persistent habits, malocclusion was the most frequently identified concern (30.7%), followed by jaw growth problems (18.9%) and speech problems (13.5%).

### Parental attitudes and practices regarding children’s oral habits, Table 4

Concerning parental attitudes and practices regarding children’s oral habits, a significant majority (92.9%, *p* < 0.0001) had never consulted a dentist about their child’s persistent habit. Out of 40 mothers (7%) who had consulted a dentist, only 16 reported that their child stopped the oral habit; the other 22 of the 40 mothers stated that dentists primarily suggested ‘only guidance’ as the treatment approach to stop oral habits. Additionally, a statistically significant proportion of mothers (37.1%, *p* < 0.0001) indicated that they would encourage their child to stop a habit if present. The most commonly chosen method for habit cessation (28.4%) was ‘frequent reminders’. Interestingly, only 16.3% of mothers observed improvements in their children’s teeth/face after habit cessation. All responses in this section showed statistically significant differences (*p* < 0.0001).

**Table 4:** Distribution and comparison of parental attitude and practice responses towards children’s oral habits.

Items of Attitude and practice	No. (%)	Χ^2^-value	*p*
Have you ever consulted a dentist about your child persistent oral habit?		404.137	< 0.0001
Yes	40 (7.1)		
No	523 (92.9)		
If yes, did the habit stop after consulting the dentist? (*n* = 43)			
Yes	16 (40)	--	--
No	24 (60)		
If yes, what treatment was suggested by the dentist?			
Only guidance was given because my child is still young	22 (55)	--	--
Reminder therapy	8 (20)		
Rewards concept	4 (10)		
Orthodontic appliance treatment (a device was used)	6 (15)		
If your child has an oral habit, did you try to help (encourage) your child to stop the habit?		206.593	< 0.0001
My child does not have a habit	315 (56.0)		
Yes	209 (37.1)		
No	39 (6.9)		
If yes, what method did you use to stop the habit?(Multiple responses) (*n* = 449)			
I did not encourage my child to stop the habit	37 (7.1)		
Frequent reminders	148 (8.4)		
Rewarding them when they stop the habit (Rewards concept)	58 (11.1)		
Punishment	16 (3.1)		
Use of appliances or restrains	57 (10.9)		
My child has no habit	178 (34.2)		
Others	27 (5.2)		
If your child had a habit, did you notice any improvement in your child’s teeth/face after the habit was stopped?			
My child did not have an oral habit	315 (56.0)	293.38	
Yes	92 (16.3)		
No	59 (16.3)		
I don’t know	97 (17.2)		

### Associations between maternal characteristics (demographics) and knowledge responses, Tables 5 and 6

Impact of Educational Status on Knowledge: Table 5 compares mothers’ knowledge responses about oral habits based on educational level. Only one item: ‘Do you think prolonged use of pacifiers or bottle feeding affect your child’s teeth?’ showed a statistically significant difference (*p* < 0.011). Mothers with less than a high school education (85.3%) and those with a high school diploma (77.2%) were more likely to agree that prolonged pacifier/bottle use negatively affects teeth than other mothers. The responses for all other items of knowledge were not statistically significantly different based on the educational status of mothers.

**Table 5:** Comparison of parental knowledge responses towards children’s oral habits about mother’s educational status.

Items of knowledge	Educational status				Χ^2^-value	*p*
	Less than high school	High school	Bachelors/Diploma	PG (Master’s or PHD)		
Do you think prolonged use of pacifiers or bottle feeding have an effect on your child’s teeth?					16.483	0.011
Yes	29 (85.3)	61 (77.2)	254 (63.3)	31 (63.3)		
No	0	5 (6.3)	52 (13.2)	10 (20.4)		
I don’t know	5 (14.7)	13 (16.5)	95 (23.7)	8 (16.3)		
Do you think that some oral habits such as (digit sucking, lip and cheek biting) can affect your child teeth?					10.168	0.118
Yes	22 (64.7)	54 (68.4)	312 (77.8)	32 (65.3)		
No	3 (8.8)	10 (12.7)	32 (8.0)	4 (8.2)		
I don’t know	9 (26.5)	15 (19.0)	57 (14.2)	13 (26.5)		
Do you think oral habits can be prevented?					4.501	0.609
Yes	22 (64.7)	59 (74.7)	312 (77.8)	39 (79.6)		
No	2 (5.9)	6 (7.6)	23 (5.7)	3 (6.1)		
I don’t know	10 (29.4)	14 (17.7)	66 (16.5)	7 (14.3)		
At what age do you think it is recommended to help a child stop a sucking habit (such as pacifier or digit sucking)					4.852	0.563
18 months	25 (73.5)	62 (78.5)	300 (74.8)	34 (69.4)		
3 years	9 (26.5)	13 (16.5)	86 (21.4)	14 (28.6)		
6 years	0	4 (5.1)	15 (3.7)	1 (2.0)		
Which aspect regarding oral habits do you think is more important and can have an effect on teeth and facial changes?					4.674	0.586
Frequency of the habit	4 (11.8)	22 (27.8)	92 (22.9)	11 (22.4)		
Duration of the habit per day	14 (41.2)	25 (31.6)	120 (29.9)	16 (32.7)		
Intensity of the force and pressure present during the habit	16 (47.1)	32 (40.5)	189 (47.1)	22 (44.9)		
Do you think the habit breaking treatment will be different according to the child’s age?					4.578	0.599
Yes	30 (88.2)	69 (87.3)	347 (86.5)	42 (85.7)		
No	0	0	10 (2.5)	0		
I don’t know	4 (11.8)	10 (12.7)	44 (11.0)	7 (14.3)		

### Impact of number of children on knowledge and attitudes

Table 6 presents a comparison of mothers’ knowledge item responses toward children’s oral habits in relation to the number of children (1–2; 3–4; or ≥ 5). Three items showed significant differences: for ‘prolonged pacifier/bottle use affecting teeth’, mothers with more than five children (79.2%, *p* < 0.0001) were more likely to agree that prolonged pacifier/bottle use affects teeth compared to mothers with fewer children. Similarly, for ‘oral habit prevention’, mothers with 1–2 children were more likely to believe oral habits are preventable (83%, *p* = 0.016). Likewise, for ‘recommended age for stopping sucking habits’, mothers with 1–2 children were more likely to approve that oral habits should be stopped by the age of 18 months (81.8%, *p* = 0.017) compared to mothers with a higher number of children. Responses to other knowledge items did not show significant differences across the three categories of the number of children, Table 6.

**Table 6:** Comparison of parental knowledge responses towards children’s oral habits regarding number of children.

Items of knowledge	Number of children			Χ^2^-value	*p*
	1 to 2	3 to 4	≥ 5		
Do you think prolonged use of pacifiers or bottle feeding have an effect on your child’s teeth?				31.062	< 0.0001
Yes	95 (54.0)	112 (64.0)	168 (79.2)		
No	25 (14.2)	27 (15.4)	15 (7.1)		
I don’t know	56 (31.8)	36 (20.6)	29 (13.7)		
Do you think that some oral habits such as (digit sucking, lip and cheek biting) can affect your child teeth?				5.645	0.227
Yes	139 (79.0)	125 (71.4)	156 (73.6)		
No	17 (9.7)	16 (9.1)	16 (7.5)		
I don’t know	20 (11.4)	34 (19.4)	40 (18.9)		
Do you think oral habits can be prevented?				12.138	0.016
Yes	146 (83.0)	129 (73.7)	157 (74.1)		
No	13 (7.4)	7 (4.0)	14 (6.6)		
I don’t know	17 (9.7)	39 (22.3)	41 (19.3)		
At what age do you think it is recommended to help a child stop a sucking habit (such as pacifier or digit sucking)				12.085	0.017
18 months	144 (81.8)	116 (66.3)	161 (75.9)		
3 years	29 (16.5)	50 (28.6)	43 (20.3)		
6 years	3 (1.7)	9 (5.1)	8 (3.8)		
Which aspect regarding oral habits do you think is more important and can have an effect on teeth and facial changes?				5.071	0.280
Frequency of the habit	34 (19.3)	42 (24.0)	53 (25.0)		
Duration of the habit per day	49 (27.8)	56 (32.0)	70 (33.0)		
Intensity of the force and pressure present during the habit	93 (52.8)	77 (44.0)	89 (42.0)		
Do you think the habit breaking treatment will be different according to the child’s age?				0.913	0.923
Yes	153 (86.9)	151 (86.3)	184 (86.8)		
No	4 (2.3)	2 (1.1)	4 (1.9)		
I don’t know	19 (10.8)	22 (12.6)	24 (11.3)		

### Associations between maternal characteristics (demographics) and attitudes responses, Tables 7 and 8

Impact of Educational Status on Attitudes/Practices: Table 7 compares mothers’ attitudes and practices towards their children’s oral habits based on educational levels. Two items showed significant differences: for, ‘If your child has an oral habit, did you try to help (encourage) your child to stop the habit?’, mothers with higher education levels were more likely to report encouraging their children to stop habits (*p* < 0.005). Also, for, ‘If your child had a habit, did you notice any improvement in your child’s teeth/face after the habit was stopped?’ mothers with higher education levels observed improvements after habit cessation (*p* < 0.028), Table 7.

**Table 7:** Distribution and comparison of parental attitude and practice responses towards their children’s oral habits in relation to mother’s educational status.

Items of attitude and practice	Educational status				Χ^2^-value	*p*
	Less than high school	High school	Bachelors/Diploma	PG (Master’s or PHD)		
Have you ever consulted a dentist about your child persistent oral habit?					0.699	0.873
Yes	2 (5.9)	5 (6.3)	33 (8.2)	3 (6.1)		
No	32 (94.1)	74 (93.7)	368 (91.8)	46 (93.9)		
If your child has an oral habit, did you try to help (encourage) your child to stop the habit?					18.481	0.005
My child does not have a habit	24 (70.6)	30 (38.0)	233 (58.1)	28 (57.1)		
Yes	8 (23.5)	38 (48.1)	143 (35.7)	20 (40.8)		
No	2 (5.9)	11 (13.9)	25 (6.2)	1 (2.0)		
If your child had a habit, did you notice any improvement in your child’s teeth/face after the habit was stopped?					18.645	0.028
My child did not have an oral habit	24 (70.6)	30 (38.0)	233 (58.1)	28 (57.1)		
Yes	4 (11.8)	21 (26.6)	62 (15.5)	5 (10.2)		
No	1 (2.9)	11 (13.9)	39 (9.7)	8 (16.3)		
I don’t know	5 (14.7)	17 (21.5)	67 (16.7)	8 (16.3)		

### Impact of number of children on attitudes/practices

Table 8 compares mothers’ attitudes and practices towards their children’s oral habits based on the number of children (1–2; 3–4; or ≥ 5). No statistically significant associations were found between the number of children and parental attitudes/practices.

**Table 8:** Distribution and comparison of parental attitude and practice responses towards their children’s oral habits in relation to number of children.

Items of Attitude and practice	Number of children			Χ^2^-value	*p*
	1 to 2	3 to 4	≥ 5		
Have you ever consulted a dentist about your child persistent oral habit?				0.697	0.706
Yes	14 (8.0)	11 (6.3)	18 (8.5)		
No	162 (92.0)	164 (93.7)	194 (91.5)		
If your child has an oral habit, did you try to help (encourage) your child to stop the habit?				6.013	0.198
My child does not have a habit	108 (61.4)	98 (56.0)	109 (51.4)		
Yes	59 (33.5)	61 (34.9)	89 (42.0)		
No	9 (5.1)	16 (9.1)	14 (6.6)		
If your child had a habit, did you notice any improvement in your child’s teeth/face after the habit was stopped?				12.187	0.058
My child did not have an oral habit	108 (61.4)	98 (56.0)	109 (51.4)		
Yes	24 (13.6)	22 (12.6)	46 (21.7)		
No	17 (9.7)	25 (14.3)	17 (8.0)		
I don’t know	27 (15.3)	30 (17.1)	40 (18.9)		

## Discussion

Deleterious oral habits can lead to impairments in speech articulation, swallowing, chewing, and sucking. Parents’ knowledge, awareness, and attitudes towards their children’s oral habits play a crucial role in establishing and maintaining oral health-related habits from infancy through early childhood [37, 39]. Parents can influence their children’s behaviors during childhood by encouraging or discouraging particular habits. Therefore, it is essential to provide parents with information concerning treatments and outcomes to ensure adequate knowledge about oral health promotion and to encourage positive attitudes and practices for effectively managing children’s oral habits [32, 40].

Our findings suggest a moderate level of awareness among recruited mothers, with the majority recognising the negative effects of prolonged pacifier/bottle use and other specific oral habits on children’s teeth (Table 3). These findings partially align with a previous cross-sectional study among a group of parents, where parents demonstrated awareness of the dangers associated with excessive pacifier use [41, 42].

In this study, results showed an increased awareness from Saudi parents regarding oral habits such as digit sucking, lip biting, and cheek biting, how they can negatively affect dental health, and how this should be prevented. However, discrepancies exist compared to other studies reporting lower knowledge and awareness levels among Saudi parents [2, 37, and 39]. This variation could be attributed to several factors, including differences in study populations, sample sizes, and methodologies. In terms of study population differences, our study’s participants could be of a higher socioeconomic level with greater access to healthcare information than other studies. Sample size dissimilarity and smaller sample sizes in other studies could affect the ability to generalise their findings. Differences in data collection methods (questionnaires vs. interviews) could also have contributed to different responses from participants.

Regarding comparisons of oral habits, our findings align with previous studies in identifying common oral habits among children. The most predominant oral habits reported in our study were nail-biting (25.5%) and bruxism (17.1%), as shown in Table 2. This differs from prior studies reporting thumb-sucking as the most common habit, followed by tongue thrust [29, 37]. Another study identified nail-biting as the most prevalent, followed by mouth-breathing, thumb-sucking, and lastly, bruxism [18]. Several factors, such as culture, socioeconomic factors, and the age of children, could have contributed to the discrepancies between our results and the results of previous studies. Certain cultural practices are likely to lead to the adoption of pacifiers and the discouragement of thumb-sucking, leading to variations in the prevalence of oral habits. Socioeconomic variations can influence access to pacifiers and the types of oral habits that develop in children from different backgrounds.

Concerning parental attitudes, this study results indicated that most mothers (over 70%) believed children should stop sucking habits by 18 months. On the other hand, evidence-based research studies indicate that detrimental changes resulting from sucking habits persist after the cessation of the habit; therefore, it has been recommended that earlier intervention could be beneficial [9]. Most participating parents in this study agreed that persistent oral habits cause malocclusion and growth problems. This is consistent with other studies reporting that a prolonged finger-sucking habit was associated with significant malocclusion criteria, including protrusion of upper incisors, anterior open bite, increased overjet, and crossbite [43]. In addition, another study reported highly statistically significant relations between malocclusion and thumb-sucking and pacifier habits [18]. Moreover, our study reveals that participating parents perceived the intensity of force and pressure exerted during the performance of the oral habit to be critical factors affecting teeth and facial growth. These results were endorsed by other studies that concluded that the duration of harmful oral habits is directly associated with damaging changes in oral structures and functions [23, 24, 26]. It has been reported that dental changes often correct themselves spontaneously if a habit stops before 5 years of age [44]. However, if dental and orofacial changes persist until the eruption of permanent anterior teeth, appropriate intervention and dental treatment should start based on the child’s development, comprehension, and ability to cooperate [45]. Habit treatment modalities include patient/parent counselling, behaviour modification techniques, myofunctional therapy, and appliance therapy (extraoral and intraoral), Table 3 [19].

Regarding parents’ educational status, we found a positive relationship between mothers’ educational status and parental knowledge and attitudes towards children’s oral habits. This was documented by mothers’ reported attempts to encourage habit cessation and observe improvements after habit stoppage, Table 7. This is in opposition to other studies that indicated that Saudi parents’ knowledge and awareness about oral health are deficient [37]. Accordingly, educational programmes tailored for parents could serve as a viable plan to enhance parents’ knowledge, attitudes, and practices toward children’s oral habits [32].

This study revealed a significant gap between knowledge and practice regarding seeking professional help. Most mothers (92.9%) in this study had never consulted a dentist about their child’s persistent habit, as shown in Table 4. This contradicts other studies that reported a higher prevalence of parents seeking regular dental care for their children [38, 42]. On the other hand, another study reported that 95% of parents only took their children to the dentist when problems arose [1]. Several factors, such as cultural differences in healthcare utilisation, dental treatment cost barriers, parents’ low socioeconomic status, a lack of higher education, and a lack of awareness about the services provided through paediatric dentists, could explain this discrepancy, Tables 5 and 7.

An intriguing finding of our study is the positive relationship between parental knowledge and attitudes towards children’s oral habits and an increasing number of children. This could be attributed to the added experience and oral health awareness mothers gain after interacting with each additional child. Conversely, other studies have found no association between knowledge and awareness of oral health and factors such as occupation or number of children in the family, Tables 6 and 8 [37].

This study demonstrates several key strengths that enhance its reliability and practical relevance. Firstly, it employed a validated, translated questionnaire to collect consistent data from 563 participants, ensuring linguistic and cultural appropriateness when examining maternal characteristics – such as education level and number of children –and their impact on knowledge and attitudes towards oral health habits. The cross-sectional design offered a comprehensive snapshot of current parental practices within the cultural context, effectively identifying gaps between knowledge and behaviours. Additionally, the study provided valuable cultural insights, shedding light on how cultural factors shape oral health practices and beliefs. Demographic insights further enriched the findings, emphasising the influence of parental characteristics on understanding and practices related to children’s oral health. Lastly, the study’s practical implications are significant, offering a foundation for targeted educational interventions to improve public health outcomes by addressing the knowledge-behaviour gap.

## Limitations

One limitation of this study is the cross-sectional design, which hinders the establishment of causal relationships between the examined variables. Another limitation is the nature of the self-reported collected data, which could have introduced bias based on social appeal. Future research employing longitudinal designs with more objective measurements of oral habits would be valuable for providing a more objective evidence base.

## Directions for future research

1. Future research should explore the reasons behind the low parental consultation rates for persistent oral habits.
2. Investigations of cultural beliefs and socioeconomic factors that influence healthcare adoption patterns concerning children’s oral health would be valuable.
3. Additional studies are required to evaluate the value of educational programmes to improve parental knowledge and awareness regarding oral habits. Such research is deemed essential for tailoring educational programmes to address the specific needs and perspectives of parents in Saudi Arabia.

## Conclusions

This study investigated parental knowledge, awareness, and attitudes regarding children’s oral habits in Riyadh, Saudi Arabia. The findings suggest a moderate level of awareness among mothers, with a positive association between educational level and knowledge about oral habits. However, a significant gap exists between knowledge and the attitudes or practices of seeking professional help for persistent habits. Overall, this study emphasises raising awareness and educating parents on the importance of early dental interventions to prevent harmful oral habits. Bridging these gaps would contribute to better oral health outcomes for children and ensure a better quality of life.

## Data Availability

The data presented in this study are available on request from the corresponding author.

## STROBE statement

Manuscript is prepared according to STROBE checklist of items that should be included in reports of observational studies. A copy of the checklist is submitted during submission.
